# Microbiota and Pathogen Proteases Modulate Type III Secretion Activity in Enterohemorrhagic Escherichia coli

**DOI:** 10.1128/mBio.02204-18

**Published:** 2018-12-04

**Authors:** Elizabeth A. Cameron, Meredith M. Curtis, Aman Kumar, Gary M. Dunny, Vanessa Sperandio

**Affiliations:** a Department of Microbiology, University of Texas Southwestern Medical Center, Dallas, Texas, USA; b Department of Biochemistry, University of Texas Southwestern Medical Center, Dallas, Texas, USA; c Department of Microbiology and Immunology, University of Minnesota, Minneapolis, Minnesota, USA; Johns Hopkins Bloomberg School of Public Health; Vanderbilt University; University of Georgia

**Keywords:** EspP, *Bacteroides*, enterohemorrhagic *E*. *coli* (EHEC), microbiota, type three secretion

## Abstract

Enteric pathogens have complex interactions with the gut microbiota. Most of what is known about them has focused on microbiota-derived metabolites or small molecules that serve as nutrients and/or signals to aid in growth or transcriptionally regulate virulence gene expression. A common virulence strategy is to express a type III secretion system (T3SS), which is a molecular syringe deployed by many Gram-negative pathogens to hijack host cell function. Enterohemorrhagic Escherichia coli (EHEC) requires its T3SS to colonize the intestinal tract and cause disease. Here we report that a prominent member of the intestinal microbiota, *Bacteroides thetaiotamicron* (*Bt*), secretes proteases that cleave the translocon of the T3SS of EHEC to enhance effector translocation into host cells. This is in contrast from an endogenous protease from EHEC itself (namely, EspP) that cleaves the translocon protein EspB in a different site to limit effector translocation. The EspB protein forms the T3SS pore in mammalian cells, and pore proteins are conserved in the T3SSs from several pathogens. This is the first demonstration of a commensal species directly processing a pathogen’s T3SS, posing a new paradigm for how the microbiota can influence the severity of disease caused by bacterial pathogens. Because T3SSs are employed by many pathogens, this phenomenon has broad implications to commensal-pathogen relationships.

## Abstract (precis)

The gut microbiota is usually regarded as providing colonization resistance against enteric pathogens. However, some pathogens evolved to thrive with the aid of certain members of the microbiota. Several Gram-negative bacteria employ type three secretion systems (T3SSs), which are molecular syringes that deliver effector proteins to host cells, hijacking host cell function. Here we show that the T3SS of enterohemorrhagic E. coli (EHEC) is cleaved by self and microbiota-derived proteases. Self-cleavage limits effector translocation, while cleavage by the microbiota member *Bacteroides thetaiotamicron* (*Bt*) exacerbates effector translocation and lesion formation on epithelial cells.

## INTRODUCTION

Upon entering the intestinal tract, pathogenic bacteria are confronted with a densely populated ecosystem saturated with bacterial competitors. The bacterial species that comprise the microbiota can significantly affect the course of disease during an infection with an intestinal pathogen in ways that we are just beginning to understand (1). Enterohemorrhagic Escherichia coli (EHEC) is an enteric pathogen that colonizes the colon and causes outbreaks of bloody diarrhea and hemolytic uremic syndrome (HUS) worldwide. It causes more than 265,000 food-borne infections and more than 30 deaths each year in the United States (2). EHEC has a remarkably low infectious dose compared to those of other intestinal pathogens. As little as 10 to 100 organisms are sufficient to cause disease (3), suggesting that EHEC has evolved particularly effective strategies to compete and even take advantage of the resident microbes it encounters in the intestinal tract. Previous studies have shown that EHEC uses microbiota-derived molecules, such as fucose (4) and succinate (5), as signals to assess the environment and control expression of its virulence factors.

**FIG 1:**
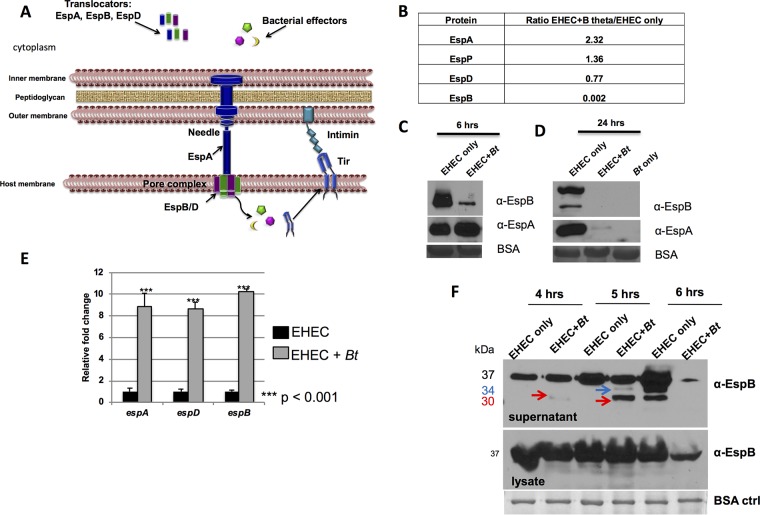
The EHEC T3SS is cleaved by self-produced and Bacteroides thetaiotaomicron (*Bt*)-produced proteases. (A) EHEC T3SS: EspA is a sheath around the needle that connects to the pore proteins EspBD, forming the translocon. Several effectors, one of which is Tir, are translocated to the host cell. Tir serves as the receptor for the bacterial adhesin intimin and promotes actin polymerization, leading to AE lesion formation. (B) Mass spectrometric analysis of culture supernatants. Data are presented as a ratio of protein present in supernatants from EHEC grown in the presence of B. thetaiotaomicron (B theta) to protein present in supernatants from EHEC grown alone. (C and D) Western blot analysis of endogenous EspAB levels in culture supernatants of EHEC alone or EHEC plus *Bt* cultured for 6 h (C) and 24 h (D) in *in vitro* culture. EspAB levels in culture supernatants were analyzed by Western blotting using anti-EspAB antibodies. BSA is used as a loading control. α-EspB, anti-EspB antibody. (E) qPCR of *espADB* operon. *espABD* transcript levels from EHEC cultures with or without *Bt* were normalized to *rpoA* levels and are expressed as fold change over the values for EHEC alone. (F) Endogenous EspB levels. *In vitro* culture supernatants and lysates were collected at 4, 5, and 6 hours, and EspB was analyzed by Western blotting. The positions of the 37-kDa uncleaved EspB, 34-kDa cleaved EspB (blue arrows), and 30-kDa cleaved EspB (red arrows) are indicated. ctrl, control.

The two main virulence factors expressed by EHEC are Shiga toxin and a type III secretion system (T3SS), which is a syringe-like apparatus used to inject bacterial effectors into the host cell that either mimic or hijack host cell function (6). The EHEC T3SS is encoded on a pathogenicity island named the locus of enterocyte effacement (LEE) and is required for EHEC to colonize the intestine and cause disease (7, 8). Three LEE-encoded proteins, EspA, EspB, and EspD, form the translocon of the EHEC T3SS: EspA forms a sheath around the needle, and EspB and EspD form the pore in the host cell membrane (9, 10) (Fig. 1A). LEE-encoded and non-LEE-encoded effector proteins are injected into host cells through the translocon and interact with host proteins, leading to actin polymerization and formation of the characteristic attaching and effacing (AE) lesion, a pedestal-like structure that cups the bacterial cell. This pedestal structure promotes tight adherence of the bacterium to host cells and leads to effacement of the intestinal microvilli (11).

Expression of the LEE is modulated by a complex regulatory cascade that incorporates environmental and microbiota- and host-derived signals (12–15). However, much of this work has focused on modulation of the LEE at the transcriptional level, with less emphasis on posttranscriptional or posttranslational regulation. Even less studied is the way in which the microbiota and microbiota-derived molecules may affect virulence factors posttranscriptionally or posttranslationally. In this study, we investigated the effect of a common human commensal, *Bacteroides thetaiotamicron* (*Bt*), on the expression of T3SS-associated proteins and their functions. The presence of *Bt* affects LEE expression at the transcriptional level (4, 5), but this interaction has not been probed further to understand more fully the relationship between commensals and pathogens during the course of an infection. Here we show that EHEC- and *Bt*-secreted proteases cleave the T3SS translocon proteins. These cleavage events differentially impact T3SS function. Cleavage by EHEC's serine protease autotransporter (SPATE), EspP, limits effector translocation to host cells. Meanwhile, processing of the T3SS by *Bt* proteases promotes effector translocation, increasing AE lesion formation on epithelial cells.

## RESULTS

### The EHEC T3SS is cleaved by EHEC-produced and *Bt*-produced proteases.

To investigate the effect of *Bacteroides thetaiotamicron* (*Bt*) on EHEC’s T3SS, secreted proteins from EHEC monoculture and EHEC+*Bt* coculture were analyzed via mass spectrometry (see Table S1 and Fig. S1 in the supplemental material). We identified 37 proteins in EHEC supernatants whose levels were significantly changed, including secreted proteases, outer membrane and iron binding proteins, enzymes, and stress proteins among others. It is noticeable that EspB and EspD, two structural components of the T3SS, were decreased in the EHEC+*Bt* coculture (Fig. 1B) with an approximately 500-fold decrease in protein levels compared to EHEC alone for EspB (Fig. 1B). After six hours of growth in the presence of *Bt*, decreased EspB was observed in supernatants compared to supernatants of EHEC cultured alone (Fig. 1C), and at 24 h, EspB was not detected (Fig. 1D). The levels of EspA were unchanged in EHEC+*Bt* coculture at six hours of growth (Fig. 1C) but decreased by 24 h (Fig. 1D). However, it was previously reported that the presence of *Bt* enhances transcription of the T3SS genes (5). Indeed, transcription of *espABD* is upregulated in the presence of *Bt* (Fig. 1E), despite protein levels in supernatants being decreased, suggesting that protein levels are being modulated posttranscriptionally. To define the timing of EspB cleavage, samples from EHEC monoculture and EHEC+*Bt* coculture were collected at 4, 5, and 6 h postinoculation (p.i.), and EspB levels in supernatants and cell lysates were analyzed. At 4 h p.i., there is little EspB cleavage, and by 5 h, EspB cleavage products are visible in the coculture condition. At 6 h, cleavage of EspB occurs in the EHEC-alone condition as well as in the presence of *Bt*, where EspB levels are severely decreased. EspB cleavage occurs after secretion and is not observed in whole-cell lysates (Fig. 1F). It is noticeable that the overall levels of EspB decrease at the 6-h time point (Fig. 1F). This is probably due to the decrease in the overall transcription of the LEE genes at this time point, which is when EHEC reaches stationary phase (16).

10.1128/mBio.02204-18.1 TABLE S1 Secreted proteins. The values for the ratio of EHEC plus *B. thetaiotaomicron/*EHEC alone are shown. Download Table S1, PDF file, 0.1 MB. Copyright © 2018 Cameron et al. 2018 Cameron et al. This content is distributed under the terms of the Creative Commons Attribution 4.0 International license.

10.1128/mBio.02204-18.4 FIG S1 Coomassie blue stain of supernatants from EHEC grown in the presence or absence of *Bt* for 6 hours. EspB is no longer present when EHEC is grown in the presence of *Bt*. Download FIG S1, PDF file, 0.2 MB. Copyright © 2018 Cameron et al. 2018 Cameron et al. This content is distributed under the terms of the Creative Commons Attribution 4.0 International license.

### Secreted proteases from EHEC and *Bt* differentially cleave the T3SS translocon components.

**FIG 2:**
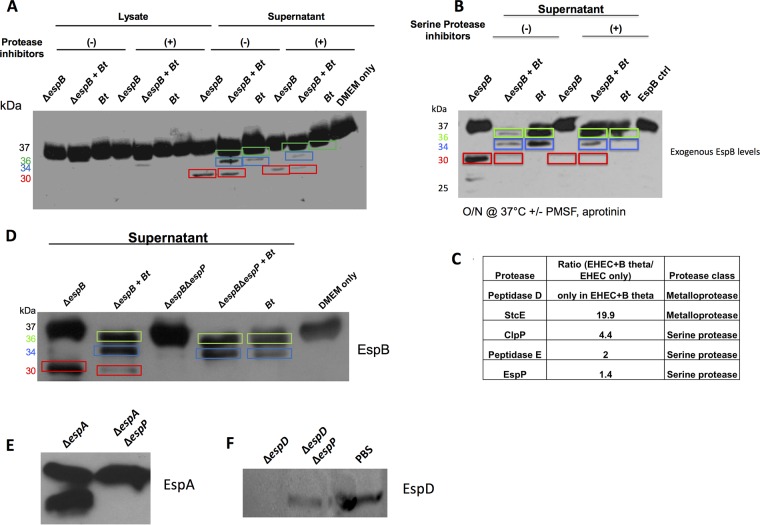
Both EHEC and *Bt* secrete proteases targeting EspB for degradation. (A) Western blot analysis of exogenous EspB. Purified rEspB-His was incubated with either the lysate or supernatant from EHEC *ΔespB* grown alone, EHEC *ΔespB* plus *Bt*, or *Bt* grown alone. Cultures were grown for 4.5 h prior to harvest. EspB-His (1 μg) was incubated at 37°C for 45 min in the presence or absence of a protease inhibitor cocktail (Sigma). (B) Exogenous EspB assay with serine protease inhibitors. rEspB-His (1 μg) was incubated with supernatants from specified cultures overnight in the presence or absence of serine protease inhibitors (PMSF, aprotinin) at 37°C. O/N, overnight. (C) Mass spectrometric analysis of proteases present in culture supernatant. Data are presented as a ratio of EHEC+*Bt*/EHEC alone. (D) Western blot analysis of exogenous EspB. Purified EspB-His was incubated with supernatants from EHEC *ΔespB* grown alone, EHEC *ΔespB* plus *Bt*, EHEC *ΔespB* Δ*espP*, EHEC *ΔespB* Δ*espP* plus *Bt*, or *Bt* grown alone. Cultures were grown for 4.5 h prior to harvest. EspB-His (1 μg) was incubated at 37°C for 45 min. DMEM medium was used as a negative control. (E) Western blot analysis of exogenous EspA. Purified EspA-His was incubated with supernatants from EHEC *ΔespA* or EHEC *ΔespA ΔespP*. (F) Western blot analysis of exogenous EspD. Purified EspD-His was incubated with supernatants from EHEC *ΔespD*, EHEC *ΔespD ΔespP*, or PBS as a negative control. Different EspB cleaved products are shown boxed in different colors: green, 36-kDa product; blue, 34-kDa product; and red, 30-kDa product.

An assay combining purified recombinant EspB (rEspB) with supernatants or lysates from mono- or cocultures of EHEC (EHEC Δ*espB* was used to eliminate confounding effects from endogenous EspB production) and *Bt* showed that cleavage of EspB by EHEC and *Bt* leads to distinct products. As expected, there was no EspB cleavage with cell lysates, confirming that EspB cleavage occurs by secreted EHEC and *Bt* proteases. Distinct rEspB cleavage products were observed with supernatants: EHEC supernatants yielded a 30-kDa rEspB, and *Bt* supernatants yielded 36-kDa and 34-kDa rEspBs (Fig. 2A). Protease inhibitors decreased the 30-kDa and 34-kDa rEspBs (Fig. 2A), confirming that these proteins were cleaved by EHEC- and *Bt*-derived proteases at different sites. It is noteworthy that cleavage of EspB is enhanced with the EHEC+*Bt* supernatants, compared to *Bt* supernatants alone (Fig. 2A and B). There are two potential explanations to these data; one is that EHEC enhances protease expression in *Bt*, the second is that both EHEC and *Bt* proteases act in a synergistic fashion. Class-specific protease inhibitors indicated that the EHEC and *Bt* proteases that yielded the 30-kDa and 34-kDa rEspBs were serine proteases (Fig. 2B). Five EHEC-secreted proteases were identified through mass spectrometry, with two of them (StcE and EspP) encoded only in the EHEC genome, while the other three are present in both EHEC and E. coli K-12 (Fig. 2C). To narrow down the candidate proteases, rEspB was incubated with supernatants from E. coli K-12, and the related AE murine pathogen Citrobacter rodentium. Supernatants from neither of them gave rise to the 30-kDa rEspB (Fig. S2A), suggesting that the protease is EHEC specific and is either StcE or EspP. EHEC Δ*stcE* still cleaved EspB in the 30-kDa form (Fig. S2B), while EHEC Δ*espP* did not (Fig. 2D), showing that StcE is not the protease cleaving EspB. Moreover, Hi-Trap-benzamidine-purified EspP (Fig. S3), specifically cleaved rEspB into the 30-kDa form (Fig. S4). Hence, EspP is the EHEC protease responsible for the 30-kDa EspB product. In addition to EspB, EspP also cleaves the EspA and EspD structural components of the T3SS (Fig. 2E and F). Through mass spectrometry on the supernatants of EHEC+*Bt* cocultures, we identified a candidate *Bt* peptidase (BT_2479) in the supernatant (Fig. S5). However, a *Bt* mutant for this gene did not lose protease activity for EspB. Mutations in six other potentially secreted candidate proteases (BT_0212, BT_1312, BT_1549, BT_1879, BT_3562, and BT_3889) in the *Bt* genome similarly did not result in loss of EspB cleavage. The *Bt* genome contains the remarkably high number of 35 putative secreted proteases (17) so it is plausible that redundant proteases are responsible for this 34-kDa cleavage product. To more directly assess the role of both EspP and *Bt* on the proteolytic cleavage of EspB, we determined the cleavage site of the 30-kDa (EspP-derived) and 34-kDa (*Bt*-derived) products of EspB via Edman sequencing. This was possible because we had determined via C-terminal His tagging of EspB that both of these cleavage events occur near the N terminus (Fig. S6A and B). Site-directed mutations of the −1 and +1 amino acid of each cleavage site were made (Fig. S6C), and these mutated *espB* alleles, *espB*Δ*Bt*site and *espB*ΔEspPsite, were used to complement Δ*espB* EHEC, and it was confirmed that the mutated EspB proteins were expressed at levels similar to those of wild-type (WT) EspB (Fig. S6D) and that they were no longer cleaved by their respective proteases. It is noteworthy that there is a decrease in EspB cleavage in the *espB*Δ*Bt*site strain in the presence of *Bt*. However, cleavage is not completely abrogated. This is probably due to the fact that the 36-kDa *Bt* cleavage site is intact. We could map only the 34-kDa site because we could not completely separate the 37- and 36-kDa forms of EspB for cleavage analyses (Fig. S6E).

10.1128/mBio.02204-18.5 FIG S2 EspP is the EHEC protease that cleaves EspB. (A) rEspB was incubated with supernatants from E. coli K-12, *ΔespB* EHEC, or a T3SS-deficient (*ΔescN)* Citrobacter rodentium strain with and without *B. thetaiotaomicron.* (B) Western blot of endogenous EspB in WT and Δ*stcE* supernatants. Download FIG S2, PDF file, 0.2 MB. Copyright © 2018 Cameron et al. 2018 Cameron et al. This content is distributed under the terms of the Creative Commons Attribution 4.0 International license.

10.1128/mBio.02204-18.6 FIG S3 Coomassie staining of HiTrap benzamidine trapping of EspP used for *espB* degradation. Δ*espB* or Δ*espB* Δ*espP* bacteria were grown in 200 ml low-glucose DMEM at 37°C and 5% CO_2_ for 4.5 h, filtered and concentrated with 10-kDa cutoff to 1 ml, 500 μl was buffer exchanged with PBS; another 500 μl was buffer exchanged with binding buffer and ran over a HiTrap benzamidine column, eluted with 7.5 ml glycine buffer (pH 3.0) into 2 ml Tris buffer, pH 9.0 → buffer exchanged with PBS to a volume of 200 μl. Incubated samples with and without 50 μg of EspB overnight at 37°C; ran on 12% SDS-PAGE and Coomassie stained Download FIG S3, PDF file, 0.3 MB. Copyright © 2018 Cameron et al. 2018 Cameron et al. This content is distributed under the terms of the Creative Commons Attribution 4.0 International license.

10.1128/mBio.02204-18.7 FIG S4 Western blot of HiTrap benzamidine trapping of EspP used for *espB* degradation. Δ*espB* or Δ*espB* Δ*espP* bacteria were grown in 200 ml low-glucose DMEM at 37°C and 5% CO_2_ for 4.5 h, filtered and concentrated with a 10-kDa cutoff to 1 ml; buffer exchanged 500 μl with PBS; buffer exchanged other 500 μl with binding buffer and ran over HiTrap benzamidine column; eluted with 7.5 ml glycine buffer (pH 3.0) into 2 ml Tris buffer, pH 9.0 → buffer exchanged with PBS to a volume of 200 μl. Incubated samples with and without 50 μg of EspB overnight at 37°C, ran on 12% SDS-PAGE, and Western blotting was performed using anti-EspB antiserum. Download FIG S4, PDF file, 0.3 MB. Copyright © 2018 Cameron et al. 2018 Cameron et al. This content is distributed under the terms of the Creative Commons Attribution 4.0 International license.

10.1128/mBio.02204-18.8 FIG S5 *Bt* proteins identified in *Bt*+EHEC coculture by mass spectrometry. Download FIG S5, PDF file, 0.2 MB. Copyright © 2018 Cameron et al. 2018 Cameron et al. This content is distributed under the terms of the Creative Commons Attribution 4.0 International license.

10.1128/mBio.02204-18.9 FIG S6 Determination of EspB proteolysis sites. (A) Schematic of pET21a-EspB-His. (B) Western blot analysis of exogenous rEspB-His using an anti-His antibody that will detect cleaved EspB only if the C-terminal His tag remains intact. (C) Schematic of site-directed mutations made to the EspP and *Bt* protease cleavage sites. (D) Western blots of whole-cell lysates collected from EHEC Δ*espB* complemented with either WT *espB, espB*Δ*Bt*site, or *espB*ΔEspPsite in pACYC184. EspB appeared to be expressed to similar levels in all three strains. RpoA was used as a control for cell numbers. (E) Western blots of supernatants of EHEC Δ*espB* complemented with WT EspB or EspBΔBtsite cloned into vector pACYC184 in the presence and absence of *Bt* using anti-EspB antisera. There is a decrease in EspB cleavage in the EspBΔBtsite strain in the presence of *Bt*. However, cleavage is not completely abrogated, this is probably due to the fact that the 36-kDa *Bt* cleavage site is intact. We could only map the 34-kDa site because we could not completely separate the 37- and 36-kDa forms of EspB for cleavage analyses. Download FIG S6, PDF file, 0.4 MB. Copyright © 2018 Cameron et al. 2018 Cameron et al. This content is distributed under the terms of the Creative Commons Attribution 4.0 International license.

### Translocon cleavage affects T3SS function.

**FIG 3:**
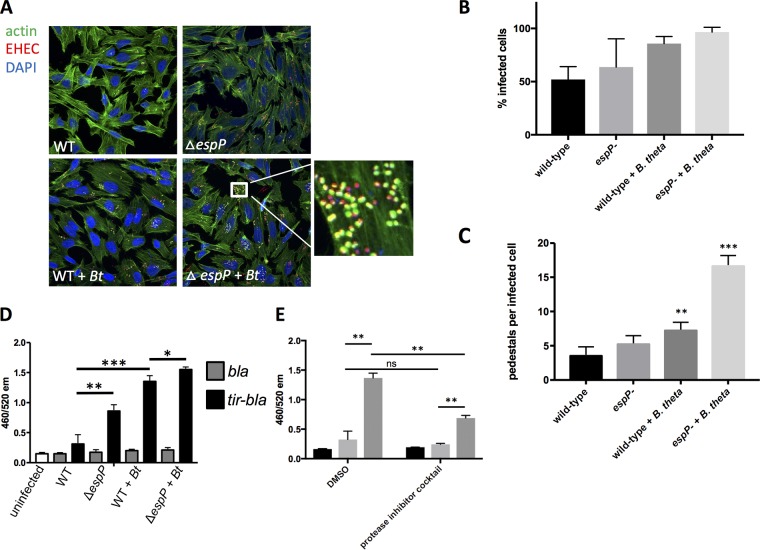
EspB cleavage affects T3SS function. (A) Fluorescein actin staining (FAS) analysis. HeLa cells were infected with mCherry-expressing EHEC (WT or Δ*espP*) with or without *Bt.* At six hours postinfection, cells were washed, fixed, and stained with DAPI and FITC-phalloidin to visualize actin. Pedestals were visualized as green puncta localized with red bacteria. (B) Quantification of percent infected cells (percentage of cells with EHEC forming pedestals). **, *P* < 0.01; ***, *P* < 0.001. (C) Quantification of number of pedestals. The number of pedestals per infected cell was determined for each field, with each field containing approximately 20 cells. The averages and standard deviation across the fields (*n* = 4) are shown. **, *P* < 0.01; ***, *P* < 0.001. (D) Tir translocation assay. A TEM-1 β-lactamase system was used to measure translocation of Tir. Reporter EHEC strains express either TEM-1 β-lactamase (*bla*) as a control or a *tir-bla* fusion that will be translocated into host cells via the T3SS. HeLa cells were infected with reporter strains (WT or Δ*espP*) with or without *Bt.* Cells were then loaded with a fluorescent β-lactam compound whose emission spectrum was altered by β-lactamase cleavage. The 460/520 emission ratio reflects the level of β-lactamase activity and therefore the level of Tir translocation into cells. *, *P* < 0.05; **, *P* < 0.01; ***, *P* < 0.001. (E) The Tir translocation assay described for panel D was performed using a WT EHEC reporter strain +/− *Bt* in the presence of a protease inhibitor cocktail or DMSO vehicle control. **, *P* < 0.01; ns, not significant.

**FIG 4:**
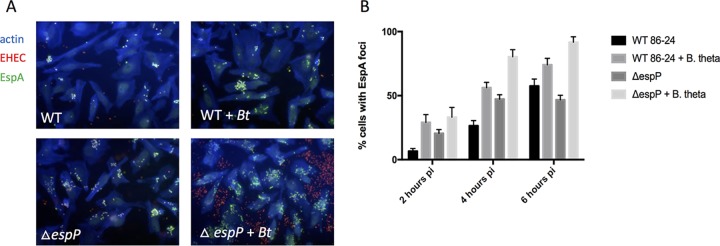
EspB proteolysis affects EspA filament formation. EspA immunofluorescence. HeLa cells were infected with mCherry-expressing EHEC (WT or Δ*espP*) with and without *Bt.* At 2, 4, and 6 h postinfection, cells were fixed, permeabilized, and stained for actin (blue) and EspA filaments (green). Representative images of the 6-h time point are shown (A) and the percentage of HeLa cells that contained EspA foci are quantified (B). The percentage of EspA-positive cells was quantified for 7 randomly selected fields containing 20 to 30 cells in each field, and the average and standard deviation are shown. The experiment was performed three times to ensure reproducibility. pi, postinoculation.

To determine the effect of translocon proteolysis on T3SS function, we assessed the ability of WT and Δ*espP* EHEC to form AE lesions on epithelial cells in the presence or absence of *Bt*. The Δ*espP* strain had a tendency to form a higher percentage of pedestal-producing cells and pedestals per infected cell than WT, but these numbers failed to reach statistical significance (Fig. 3A to C). As previously reported, the presence of *Bt* significantly increased pedestal formation by WT EHEC (5), and this was further increased in the Δ*espP* strain (Fig. 3A to C). The T3SS translocon protein EspA forms a sheath around the T3SS needle (Fig. 1A) that can be visualized by fluorescence microscopy using an anti-EspA antiserum, and is essential for effector translocation and AE lesion formation (18).The levels of EspA during infection of epithelial cells were elevated in Δ*espP* strain compared to the WT strain both in the presence and absence of *Bt*, and the presence of *Bt* further elevated EspA filaments in both strains (Fig. 4). A similar pattern was observed when translocation of the T3SS effector Tir was measured. Tir translocation was again elevated in the Δ*espP* strain in the presence and absence of *Bt*, and the presence of *Bt* further enhanced Tir translocation in both strains (Fig. 3D). Moreover, protease inhibitors decreased Tir translocation by the WT in the presence of *Bt* (Fig. 3E). Again, although inhibition of proteases decreased Tir translocation in the presence of *Bt*, it did not restore it to the levels of translocation in the absence of *Bt*. This is probably due to the transcriptional activation of *espB* expression in the presence of *Bt* due to sensing of *Bt*-produced succinate signaling (Fig. 1E) (5). These data show that *Bt* increases T3S function both transcriptionally and posttranslationally. Consequently, the EHEC protease EspP limits, while *Bt* proteases enhance effector translocation and AE lesion formation through differential cleavage of T3SS translocon components.

## DISCUSSION

Enteric pathogens interface with a dense and highly adapted gut microbiota. The microbiota is regarded as promoting colonization resistance to pathogens (19–22), and the observation that antibiotic-treated and germfree mice are more susceptible to enteric pathogens supports this hypothesis (23–29). However, certain microbiotas may lead to pathogen expansion or enhanced virulence (5, 20, 30–32). It is noteworthy that different murine microbiotas determine susceptibility to Citrobacter rodentium. Microbiota transplantation from susceptible mouse strains to nonsusceptible animals induced susceptibility to infection. Conversely, microbiota transplantation from resistant to susceptible animals led to resistance (33, 34). It has also been reported that susceptibility of Swedish adults to Campylobacter jejuni infections differed depending on the species composition of their microbiota (35). We are starting to unravel the mechanisms by which different microbiota membership can either offer resistance or aid invading pathogens.

Here we demonstrate that EHEC and a common member of the gut microbiota, Bacteroides thetaiotaomicron (*Bt*), secrete proteases that cleave structural components of the EHEC T3SS. Previous studies have demonstrated that members of the microbiota can have a significant impact on colonization and virulence gene expression by intestinal pathogens (4, 5, 31, 32). However, the majority of these studies have focused on microbiota-derived metabolites or small molecules that serve as nutrients and/or signals to aid in growth or transcriptionally regulate virulence gene expression. The role of inflammation and/or respiration in the arms race between pathogens and microbiota toward niche colonization has also been extensively documented (36–38). To our knowledge, this is the first demonstration that a protease produced by a commensal bacterium can directly cleave a pathogen’s T3SS, altering its function. It has been previously demonstrated that *Bt-*derived signals modulate transcription of the locus that encodes the EHEC T3SS (5) so it is clear that the interaction between this commensal and EHEC is multifaceted.

T3SSs are expressed by many Gram-negative bacteria and are a central virulence factor for several important human pathogens, including *Salmonella, Yersinia, Shigella,* and others. There is structural homology between the translocon components of T3SSs of different species, with EspB having homologs in many pathogens (39). EspB is a component of the T3SS pore formed in the host cell (10). However, how this pore assembles in the eukaryotic membrane is largely unknown. Our data suggest that maybe a cleavage event, such as the one promoted by *Bt*, may facilitate pore formation. There is precedence for this in the assembly of the protective antigen (PA) from the anthrax toxin, where a host-mediated cleavage of PA promotes PA oligomerization and pore formation (40). Therefore, proteolytic cleavage by microbiota-derived or endogenous proteases may be a factor broadly affecting T3SSs. This presents a new paradigm for how the microbiota may shape disease progression by pathogens.

## MATERIALS AND METHODS

### Strains, plasmids, and culture conditions.

Strains and plasmids used in this study are listed in Table S2 in the supplemental material. Bacteroides thetaiotaomicron (*Bt*) VPI-5482 was routinely grown anaerobically overnight at 37°C in TYG medium (41). Where indicated, *Bt* cultures were concentrated 10× prior to use to more closely mimic the natural ratios of EHEC:*Bt* in the gut. WT EHEC O157:H7 strain 86-24 (42) and its isogenic mutants were routinely grown in LB. In experiments where it was desired that the type III secretion system (T3SS) be expressed, DMEM low glucose (DMEM LG), defined as DMEM with 1 g/liter glucose, was used, as these conditions have been shown to induce the T3SS (43). Anaerobic growth was performed using the GasPak EZ anaerobe container system (Becton Dickinson). HeLa cells were routinely cultured in complete DMEM (cDMEM), defined as 4.5 g/liter glucose DMEM plus 10% FBS plus penicillin/streptomycin/glutamine.

10.1128/mBio.02204-18.2 TABLE S2 Strains and plasmids used in this study. Download Table S2, PDF file, 0.3 MB. Copyright © 2018 Cameron et al. 2018 Cameron et al. This content is distributed under the terms of the Creative Commons Attribution 4.0 International license.

### Recombinant DNA techniques.

All primers used in mutant and plasmid construction can be found in Table S3.

10.1128/mBio.02204-18.3 TABLE S3 Primers used in this study. Download Table S3, PDF file, 0.2 MB. Copyright © 2018 Cameron et al. 2018 Cameron et al. This content is distributed under the terms of the Creative Commons Attribution 4.0 International license.

### Construction of deletion mutants.

Isogenic mutants of EHEC 86-24 were created using the λ red recombination technique (44), using pKD4 to create the deletion PCR products and pCP20 to resolve the insertions. Deletions were confirmed by sequencing.

### Construction of vectors for protein expression.

The open reading frames of *espABD* were cloned into pET expression vectors using standard restriction digestion and ligation methods. Protein expression and purification methods are discussed below under “Exogenous rEspB proteolysis assay.”

### Construction of vectors for translocation assay.

For the *bla* control plasmid, the ribosome binding site and coding sequence of the *bla* gene were amplified from the pKD3 plasmid. To construct the *tir-bla* fusion, the ribosome binding site and coding region of *tir* (minus the stop codon) were amplified via PCR from the EHEC genome and fused to the coding region (minus the signal peptide) of the *bla* gene via soe PCR. The *bla* and *tir-bla* inserts were cloned under the control of the pBAD promoter in the pBAD33 vector using the NEB Gibson Assembly cloning kit according to the manufacturer’s instructions. Constructs were transformed into E. coli DH5, and candidate clones were screened and confirmed by sequencing. Sequence-confirmed constructs were then transformed into the desired strain of EHEC.

### Construction of vectors for *espB* protease site mutagenesis.

*espB* was cloned into the E. coli pACYC184 expression vector under the tet promoter using the NEB Gibson Assembly cloning kit according to the manufacturer’s instructions. Using this construct as a template, primers were designed to alter the −1 and +1 amino acids that surround the protease cleavage sites for EspP and one of the *Bt* proteases. Primers were designed according to the specifications outlined for the NEB Q5 site-directed mutagenesis kit. For the *Bt* (34-kDa) site, amino acids L31 and S32 were mutated to A, for the EspP (30-kDa) site, amino acid A80 was mutated to G and V81 was mutated to A according to the manufacturer’s instructions. Constructs were transformed into E. coli DH5, and candidate clones were screened by sequencing. Sequence-confirmed constructs were then transformed into the desired strain of EHEC. Protein expression was confirmed by Western blotting for EspB.

### qPCR.

For *in vitro* experiments, cultures were grown to late log phase in DMEM under anaerobic conditions (6 h). RNA was extracted using the RiboPure Bacteria isolation kit according to the manufacturer’s protocols (Ambion). The primers used for quantitative reverse transcription-PCR (qRT-PCR) (Table S2) were validated for amplification efficiency and template specificity. qRT-PCR was performed as previously described (14) in a one-step reaction using an ABI 7500 sequence detection system (Applied Biosystems). Data were collected using the ABI Sequence Detection 1.2 software (Applied Biosystems). *C_T_* values for genes of interest were normalized to the values for the *rpoA* endogenous control gene and analyzed using the comparative critical threshold (*C_T_*) method. *espABD* gene expression was presented as fold changes over the expression level of WT EHEC cultured alone. The Student’s unpaired *t* test was used to determine statistical significance.

### Mass spectrometry of secreted proteome.

EHEC was grown in LG DMEM in the presence or absence of *Bt* for 6 h, anaerobically. For total protein identification and quantification, concentrated supernatants were loaded onto SDS-polyacrylamide gel and run just until samples entered the resolving gel. The sample was extracted from the resolving gel and submitted for LC-MS/MS analysis to the UT-Southwestern proteomics core. Spectral counts were normalized to total protein for each sample and expressed as a ratio as relative quantity in EHEC alone versus EHEC+*Bt*.

### Detection of endogenous EspB cleavage products.

Overnight cultures of EHEC and *Bt* were washed, and EHEC was resuspended to 1× while *Bt* was resuspended to 10× to mimic ratios in the gut. LG DMEM (25 ml) was inoculated 1:100 with EHEC with and without *Bt*. The cultures were incubated anaerobically for 4, 5, or 6 h. At the indicated time point, cells were spun and pellet was lysed in 8 M urea lysis buffer overnight for whole-cell lysate samples. Supernatants were collected and filtered through a 0.22-μm filter, and a protease inhibitor cocktail was added. BSA (0.8 mg/ml) was added to supernatants prior to concentrating to serve as a concentration/loading control. Supernatants were then concentrated 125× using an Amicon concentrator (Millipore) with a 10-kDa-molecular-weight cutoff. Lysate and supernatant samples were mixed with SDS-PAGE loading buffer, boiled, and run on 12% SDS-PAGE gels. Protein was transferred to a PVDF membrane, and protein was detected using a rabbit anti-EspB polyclonal antibody (1:10,000) and a goat anti-rabbit HRP-conjugated secondary antibody (1:20,000). Blots were developed using ECL reagents according to the manufacturer’s instructions.

### Exogenous rEspB proteolysis assay.

pET expression vectors containing the open reading frames of *espA*, *espB*, and *espD* were transformed into E. coli BL21. Protein expression was induced with isopropyl-β-D-thiogalactopyranoside (IPTG), and proteins were purified using gravity nickel column purification. Proteins were concentrated using Amicon concentrators and were buffer exchanged into PBS (EspA and EspD) or 20 mM HEPES, 100 mM NaCl (pH 8.0) plus 10% glycerol (EspB). Supernatants were prepared by inoculating low-glucose (1 g/liter) DMEM 1:100 with overnight cultures of the indicated strains. Cultures were grown anaerobically for 6 h before cells were pelleted, and the supernatant was collected and filtered. Supernatants were concentrated 100× using 10-kDa-molecular-weight cutoff Amicon concentrators (Millipore). 60 to 100 ng of recombinant protein (varied by experiment) was combined with 150 μl concentrated supernatant and incubated at 37°C for 45 min or overnight (16 h). Samples were mixed with SDS-PAGE loading buffer to stop the proteolysis reaction and SDS-PAGE and Western blotting to visualize EspB were performed as described above for the endogenous EspB cleavage assay.

### EspB N-terminal Edman sequencing.

An exogenous EspB proteolysis assay was performed as described using supernatants from EHEC Δ*espB* and WT *Bt* to generate EspB proteolysis products. Reactions were run on a 12% SDS-PAGE gel to resolve EspB proteolysis products and blotted onto a PVDF membrane using CAPS blotting buffer as specified by the UC-Davis Proteomics Core protocol for preparing Edman sequencing samples. The membranes were then stained with Coomassie blue and destained before drying. Desired bands were cut out from the dried membrane and sent to the UC-Davis proteomics core for N-terminal Edman sequencing.

### Fluorescent actin staining (FAS) assay.

Fluorescein actin staining assays were performed as described previously (45). Briefly, HeLa cells were grown on coverslips in 12-well culture plates in cDMEM at 37°C and 5% CO2 overnight to 80% confluence. The wells were washed with PBS and replaced with low-glucose DMEM supplemented with 10% FBS. mCherry-expressing EHEC was grown standing overnight in LB, and *Bt* cultures were grown overnight anaerobically in TYG medium. Before infection, *Bt* cultures were concentrated 10× to mimic ratios in the intestinal tract. Bacterial cultures were then diluted 1:100 to infect HeLa cells for 6 h at 37°C and 5% CO2, with media being removed and replaced at 3 h postinfection. After a 6-h infection, the coverslips were washed, fixed, permeabilized, and treated with fluorescein isothiocyanate (FITC)-labeled phalloidin to visualize actin accumulation and mounted on slides with ProLong gold antifade mountant with DAPI to visualize host nuclei. Samples were visualized with a Zeiss LSM880 confocal microscope. The percent infected cells (percentage of cells containing pedestals) and pedestals per infected cell were determined for each field with each field containing approximately 20 cells. The averages and standard deviation across the fields (*n* = 4) are displayed for one experiment.

### EspA immunofluorescence.

HeLa cell infections for EspA filament staining were performed as described above for the FAS assay except that coverslips were washed and fixed at 2, 4, and 6 h postinfection. After fixing and permeabilizing, cells were treated with phalloidin–Alexa Fluor 350 to visualize host actin. Cells were then treated with 1:1,000 dilution of polyclonal anti-EspA antisera in PBS for 15 min at room temperature. Cells were then washed and stained with anti-rabbit FITC-conjugated secondary antibody (Sigma) diluted 1:1,000 in PBS for 15 min. Coverslips were mounted on slides with ProLong gold antifade and visualized with a Zeiss LSM880 confocal microscope. The percent HeLa cells with EspA-positive bacteria attached was quantified for 7 randomly selected fields containing 20 to 30 cells each, and the average and standard deviation values for the fields were calculated. The experiment was performed three times to ensure reproducibility, but the data shown are from a single experiment due to interexperimental variability.

### Translocation assay.

*bla* and *tir-bla* reporter constructs were created as described above and transformed into the desired strains of EHEC. To perform the assay, HeLa cells were seeded in at 1 × 10^4^ cells per well into a black-walled, clear-bottom 96-well plate 48 h prior to infection in cDMEM. Overnight cultures of bacterial strains (WT or Δ*espP* EHEC with and without B. thetaiotaomicron) were subcultured 1:20 into 1.5-ml DMEM LG plus 5% FBS in a 12-well plate. Cultures were grown for 3 h at 37°C in 5% CO_2_. The medium was removed from HeLa cells, cells were washed once with DMEM LG, and then 100 μl DMEM LG plus 5% FBS was added to each well. HeLa cells were infected with 15 μl of the pregrown bacterial cultures and incubated at 37°C in 5% CO_2_ for 30 min. Blank (no HeLa cells) and uninfected cells were included as controls, and each experimental condition was run in triplicate. Reporter constructs were then induced by adding 0.2% arabinose (final concentration) and incubating for 1 h. The medium was then removed from the cells, cells were washed once with HBSS, and cells were loaded with freshly prepared CCF2/AM substrate according to the manufacturer’s instructions using the GeneBLAzer *in vivo* detection kit (Invitrogen). Results were read on a FLUOstar Optima microplate reader (BMG Labtech): readings were collected from the bottom, exciting at 405 nm and reading emission at 460 nm and 520 nm. Gain was set to 10% of blank (no HeLa cells) wells. For each well, the ratio of 460/520 nm emission was calculated, and then the average and standard deviations for the three replicate wells were calculated.

## References

[1] Baumler AJ, Sperandio V 2016 Interactions between the microbiota and pathogenic bacteria in the gut. Nature 535:85–93. doi:10.1038/nature18849. 27383983 PMC5114849

[2] Scallan E, Hoekstra RM, Angulo FJ, Tauxe RV, Widdowson MA, Roy SL, Jones JL, Griffin PM 2011 Foodborne illness acquired in the United States–major pathogens. Emerg Infect Dis 17:7–15. doi:10.3201/eid1701.091101p1. 21192848 PMC3375761

[3] Tuttle J, Gomez T, Doyle MP, Wells JG, Zhao T, Tauxe RV, Griffin PM 1999 Lessons from a large outbreak of Escherichia coli O157:H7 infections: insights into the infectious dose and method of widespread contamination of hamburger patties. Epidemiol Infect 122:185–192. doi:10.1017/S0950268898001976. 10355781 PMC2809605

[4] Pacheco AR, Curtis MM, Ritchie JM, Munera D, Waldor MK, Moreira CG, Sperandio V 2012 Fucose sensing regulates bacterial intestinal colonization. Nature 492:113–117. doi:10.1038/nature11623. 23160491 PMC3518558

[5] Curtis MM, Hu Z, Klimko C, Narayanan S, Deberardinis R, Sperandio V 2014 The gut commensal Bacteroides thetaiotaomicron exacerbates enteric infection through modification of the metabolic landscape. Cell Host Microbe 16:759–769. doi:10.1016/j.chom.2014.11.005. 25498343 PMC4269104

[6] Kaper JB, Nataro JP, Mobley HL 2004 Pathogenic Escherichia coli. Nat Rev Microbiol 2:123–140. doi:10.1038/nrmicro818. 15040260

[7] McDaniel TK, Jarvis KG, Donnenberg MS, Kaper JB 1995 A genetic locus of enterocyte effacement conserved among diverse enterobacterial pathogens. Proc Natl Acad Sci U S A 92:1664–1668. doi:10.1073/pnas.92.5.1664. 7878036 PMC42580

[8] Jarvis KG, Giron JA, Jerse AE, McDaniel TK, Donnenberg MS, Kaper JB 1995 Enteropathogenic Escherichia coli contains a putative type III secretion system necessary for the export of proteins involved in attaching and effacing lesion formation. Proc Natl Acad Sci U S A 92:7996–8000. doi:10.1073/pnas.92.17.7996. 7644527 PMC41273

[9] Garmendia J, Frankel G, Crepin VF 2005 Enteropathogenic and enterohemorrhagic Escherichia coli infections: translocation, translocation, translocation. Infect Immun 73:2573–2585. doi:10.1128/IAI.73.5.2573-2585.2005. 15845459 PMC1087358

[10] Ide T, Laarmann S, Greune L, Schillers H, Oberleithner H, Schmidt MA 2001 Characterization of translocation pores inserted into plasma membranes by type III-secreted Esp proteins of enteropathogenic Escherichia coli. Cell Microbiol 3:669–679. doi:10.1046/j.1462-5822.2001.00146.x. 11580752

[11] Campellone KG, Leong JM 2003 Tails of two Tirs: actin pedestal formation by enteropathogenic E. coli and enterohemorrhagic E. coli O157:H7. Curr Opin Microbiol 6:82–90. doi:10.1016/S1369-5274(03)00005-5. 12615225

[12] Sperandio V, Torres AG, Jarvis B, Nataro JP, Kaper JB 2003 Bacteria-host communication: the language of hormones. Proc Natl Acad Sci U S A 100:8951–8956. doi:10.1073/pnas.1537100100. 12847292 PMC166419

[13] Clarke MB, Hughes DT, Zhu C, Boedeker EC, Sperandio V 2006 The QseC sensor kinase: a bacterial adrenergic receptor. Proc Natl Acad Sci U S A 103:10420–10425. doi:10.1073/pnas.0604343103. 16803956 PMC1482837

[14] Hughes DT, Clarke MB, Yamamoto K, Rasko DA, Sperandio V 2009 The QseC adrenergic signaling cascade in enterohemorrhagic E. coli (EHEC). PLoS Pathog 5:e1000553. doi:10.1371/journal.ppat.1000553. 19696934 PMC2726761

[15] Reading NC, Rasko DA, Torres AG, Sperandio V 2009 The two-component system QseEF and the membrane protein QseG link adrenergic and stress sensing to bacterial pathogenesis. Proc Natl Acad Sci U S A 106:5889–5894. doi:10.1073/pnas.0811409106. 19289831 PMC2667056

[16] Sperandio V, Mellies JL, Nguyen W, Shin S, Kaper JB 1999 Quorum sensing controls expression of the type III secretion gene transcription and protein secretion in enterohemorrhagic and enteropathogenic Escherichia coli. Proc Natl Acad Sci U S A 96:15196–15201. doi:10.1073/pnas.96.26.15196. 10611361 PMC24796

[17] Xu J, Bjursell MK, Himrod J, Deng S, Carmichael LK, Chiang HC, Hooper LV, Gordon JI 2003 A genomic view of the human-Bacteroides thetaiotaomicron symbiosis. Science 299:2074–2076. doi:10.1126/science.1080029. 12663928

[18] Knutton S, Rosenshine I, Pallen MJ, Nisan I, Neves BC, Bain C, Wolff C, Dougan G, Frankel G 1998 A novel EspA-associated surface organelle of enteropathogenic Escherichia coli involved in protein translocation into epithelial cells. EMBO J 17:2166–2176. doi:10.1093/emboj/17.8.2166. 9545230 PMC1170561

[19] Sassone-Corsi M, Raffatellu M 2015 No vacancy: how beneficial microbes cooperate with immunity to provide colonization resistance to pathogens. J Immunol 194:4081–4087. doi:10.4049/jimmunol.1403169. 25888704 PMC4402713

[20] Cameron EA, Sperandio V 2015 Frenemies: signaling and nutritional integration in pathogen-microbiota-host interactions. Cell Host Microbe 18:275–284. doi:10.1016/j.chom.2015.08.007. 26355214 PMC4567707

[21] Pacheco AR, Sperandio V 2015 Enteric pathogens exploit the microbiota-generated nutritional environment of the gut. Microbiol Spectr 3(3). doi:10.1128/microbiolspec.MBP-0001-2014. PMC5070792 26185079

[22] Bohnhoff M, Drake BL, Miller CP 1954 Effect of streptomycin on susceptibility of intestinal tract to experimental Salmonella infection. Proc Soc Exp Biol Med 86:132–137. doi:10.3181/00379727-86-21030. 13177610

[23] Ferreira RB, Gill N, Willing BP, Antunes LC, Russell SL, Croxen MA, Finlay BB 2011 The intestinal microbiota plays a role in Salmonella-induced colitis independent of pathogen colonization. PLoS One 6:e20338. doi:10.1371/journal.pone.0020338. 21633507 PMC3102097

[24] Nardi RM, Silva ME, Vieira EC, Bambirra EA, Nicoli JR 1989 Intragastric infection of germfree and conventional mice with Salmonella typhimurium. Braz J Med Biol Res 22:1389–1392. 2700668

[25] Sekirov I, Tam NM, Jogova M, Robertson ML, Li Y, Lupp C, Finlay BB 2008 Antibiotic-induced perturbations of the intestinal microbiota alter host susceptibility to enteric infection. Infect Immun 76:4726–4736. doi:10.1128/IAI.00319-08. 18678663 PMC2546810

[26] Sprinz H, Kundel DW, Dammin GJ, Horowitz RE, Schneider H, Formal SB 1961 The response of the germfree guinea pig to oral bacterial challenge with Escherichia coli and Shigella flexneri. Am J Pathol 39:681–695. 13915950 PMC1942415

[27] Wlodarska M, Willing B, Keeney KM, Menendez A, Bergstrom KS, Gill N, Russell SL, Vallance BA, Finlay BB 2011 Antibiotic treatment alters the colonic mucus layer and predisposes the host to exacerbated Citrobacter rodentium-induced colitis. Infect Immun 79:1536–1545. doi:10.1128/IAI.01104-10. 21321077 PMC3067531

[28] Zachar Z, Savage DC 1979 Microbial interference and colonization of the murine gastrointestinal tract by Listeria monocytogenes. Infect Immun 23:168–174. 106003 10.1128/iai.23.1.168-174.1979 PMC550704

[29] Kamada N, Kim YG, Sham HP, Vallance BA, Puente JL, Martens EC, Nunez G 2012 Regulated virulence controls the ability of a pathogen to compete with the gut microbiota. Science 336:1325–1329. doi:10.1126/science.1222195. 22582016 PMC3439148

[30] Yurist-Doutsch S, Arrieta MC, Vogt SL, Finlay BB 2014 Gastrointestinal microbiota-mediated control of enteric pathogens. Annu Rev Genet 48:361–382. doi:10.1146/annurev-genet-120213-092421. 25251855

[31] Ferreyra JA, Wu KJ, Hryckowian AJ, Bouley DM, Weimer BC, Sonnenburg JL 2014 Gut microbiota-produced succinate promotes C. difficile infection after antibiotic treatment or motility disturbance. Cell Host Microbe 16:770–777. doi:10.1016/j.chom.2014.11.003. 25498344 PMC4859344

[32] Ng KM, Ferreyra JA, Higginbottom SK, Lynch JB, Kashyap PC, Gopinath S, Naidu N, Choudhury B, Weimer BC, Monack DM, Sonnenburg JL 2013 Microbiota-liberated host sugars facilitate post-antibiotic expansion of enteric pathogens. Nature 502:96–99. doi:10.1038/nature12503. 23995682 PMC3825626

[33] Ghosh S, Dai C, Brown K, Rajendiran E, Makarenko S, Baker J, Ma C, Halder S, Montero M, Ionescu VA, Klegeris A, Vallance BA, Gibson DL 2011 Colonic microbiota alters host susceptibility to infectious colitis by modulating inflammation, redox status, and ion transporter gene expression. Am J Physiol Gastrointest Liver Physiol 301:G39–G49. doi:10.1152/ajpgi.00509.2010. 21454446

[34] Willing BP, Vacharaksa A, Croxen M, Thanachayanont T, Finlay BB 2011 Altering host resistance to infections through microbial transplantation. PLoS One 6:e26988. doi:10.1371/journal.pone.0026988. 22046427 PMC3203939

[35] Kampmann C, Dicksved J, Engstrand L, Rautelin H 2015 Composition of human faecal microbiota in resistance to Campylobacter infection. Clin Microbiol Infect 22:e1–61.e8. doi:10.1016/j.cmi.2015.09.004. 26369602

[36] Winter SE, Thiennimitr P, Winter MG, Butler BP, Huseby DL, Crawford RW, Russell JM, Bevins CL, Adams LG, Tsolis RM, Roth JR, Baumler AJ 2010 Gut inflammation provides a respiratory electron acceptor for Salmonella. Nature 467:426–429. doi:10.1038/nature09415. 20864996 PMC2946174

[37] Winter SE, Winter MG, Xavier MN, Thiennimitr P, Poon V, Keestra AM, Laughlin RC, Gomez G, Wu J, Lawhon SD, Popova IE, Parikh SJ, Adams LG, Tsolis RM, Stewart VJ, Baumler AJ 2013 Host-derived nitrate boosts growth of E. coli in the inflamed gut. Science 339:708–711. doi:10.1126/science.1232467. 23393266 PMC4004111

[38] Faber F, Thiennimitr P, Spiga L, Byndloss MX, Litvak Y, Lawhon S, Andrews-Polymenis HL, Winter SE, Bäumler AJ 2017 Respiration of microbiota-derived 1,2-propanediol drives Salmonella expansion during colitis. PLoS Pathog 13:e1006129. doi:10.1371/journal.ppat.1006129. 28056091 PMC5215881

[39] Deng W, Marshall NC, Rowland JL, McCoy JM, Worrall LJ, Santos AS, Strynadka NCJ, Finlay BB 2017 Assembly, structure, function and regulation of type III secretion systems. Nat Rev Microbiol 15:323–337. doi:10.1038/nrmicro.2017.20. 28392566

[40] Young JA, Collier JR 2002 Attacking anthrax. Sci Am 286:48–50, 54–49. doi:10.1038/scientificamerican0302-48. 11857900

[41] Betian HG, Linehan BA, Bryant MP, Holdeman LV 1977 Isolation of a cellulotytic Bacteroides sp. from human feces. Appl Environ Microbiol 33:1009–1010. 869523 10.1128/aem.33.4.1009-1010.1977 PMC170812

[42] Griffin PM, Ostroff SM, Tauxe RV, Greene KD, Wells JG, Lewis JH, Blake PA 1988 Illnesses associated with Escherichia coli O157:H7 infections. A broad clinical spectrum. Ann Intern Med 109:705–712. doi:10.7326/0003-4819-109-9-705. 3056169

[43] Njoroge JW, Nguyen Y, Curtis MM, Moreira CG, Sperandio V 2012 Virulence meets metabolism: Cra and KdpE gene regulation in enterohemorrhagic Escherichia coli. mBio 3:e00280-12. doi:10.1128/mBio.00280-12. 23073764 PMC3482499

[44] Datsenko KA, Wanner BL 2000 One-step inactivation of chromosomal genes in Escherichia coli K-12 using PCR products. Proc Natl Acad Sci U S A 97:6640–6645. doi:10.1073/pnas.120163297. 10829079 PMC18686

[45] Knutton S, Baldwin T, Williams PH, McNeish AS 1989 Actin accumulation at sites of bacterial adhesion to tissue culture cells: basis of a new diagnostic test for enteropathogenic and enterohemorrhagic Escherichia coli. Infect Immun 57:1290–1298. 2647635 10.1128/iai.57.4.1290-1298.1989 PMC313264

