# Supplementary material for: Microbiota and Pathogen Proteases Modulate Type III Secretion Activity in Enterohemorrhagic Escherichia coli
Source: mBio. 2018 Dec 4;9(6):e02204-18. doi: 10.1128/mBio.02204-18 (PMC6282197; doi:10.1128/mBio.02204-18)
Supplement: TABLE S1 [file mbo006184200st1.pdf]

Table S1. **Secreted Proteins: EHEC+*B. theta*/EHEC alone**

Both	Ratio EHEC+B theta/EHEC only
ATP Synthase Gamma	55.75
H-protein	51.59
StcE	19.88
Lipoamide dehydrogenase	17.57
6-phosphogluconolactonase	14.02
Outer membrane protein Tsx	12.93
Tryptophan tRNA synthetase	10.85
Aspartate ammonia-lyase (aspA)	7.53
terB (stress response)	7.3
Enolase	5.48
Beta-ketoacyl-acyl carrier protein	5.18
Leucine-specific binding protein	5.08
ClpP, serine protease	4.41
Glucose-specific PTS system IIA component	4.28
Stx2b subunit	4.16
Ferric enterobactin binding protein	3.9
Fructose-1,6-bisphosphate aldolase	3.82
2-amino-3-ketobutyrate coA ligase	3.51
Superoxide dismutase (SodB)	2.92
Serine hydroxymethyltransferase	2.71
Phosphoglycerate kinase	2.52
EspA	2.32
TerD (stress)	2.28
Triosephosphate isomerase	2.09
Aspartate aminotransferase	1.93
TerE	1.91
OmpC	1.84
EspP	1.36
EspD	0.77
rplO, 50S ribosomal subunit	0.56
single-strand DNA binding protein (ssb)	0.54
lpp, outer membrane lipoprotein	0.54
30S subunit protein S9	0.39
Recomb. assoc. protein (putative exonuclease)	0.26
Ribosomal protein S12	0.16
Ribose-5-phosphate isomerase	0.13
EspB	0.002
