# Supplementary material for: Microbiota and Pathogen Proteases Modulate Type III Secretion Activity in Enterohemorrhagic Escherichia coli
Source: mBio. 2018 Dec 4;9(6):e02204-18. doi: 10.1128/mBio.02204-18 (PMC6282197; doi:10.1128/mBio.02204-18)
Supplement: FIG S1 [file mbo006184200sf1.pdf]

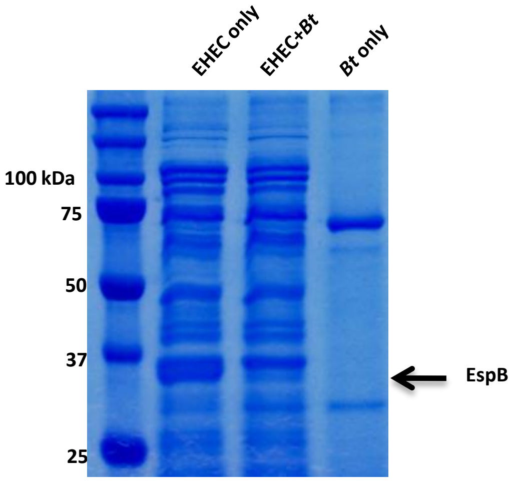

Fig.S1. Coomassie blue stain of supernatants from EHEC grown in the presence or absence of *Bt* for 6 hr. EspB is no longer present when EHEC is grown in the presence of *Bt*.
