# Supplementary material for: Microbiota and Pathogen Proteases Modulate Type III Secretion Activity in Enterohemorrhagic Escherichia coli
Source: mBio. 2018 Dec 4;9(6):e02204-18. doi: 10.1128/mBio.02204-18 (PMC6282197; doi:10.1128/mBio.02204-18)
Supplement: FIG S2 [file mbo006184200sf2.pdf]

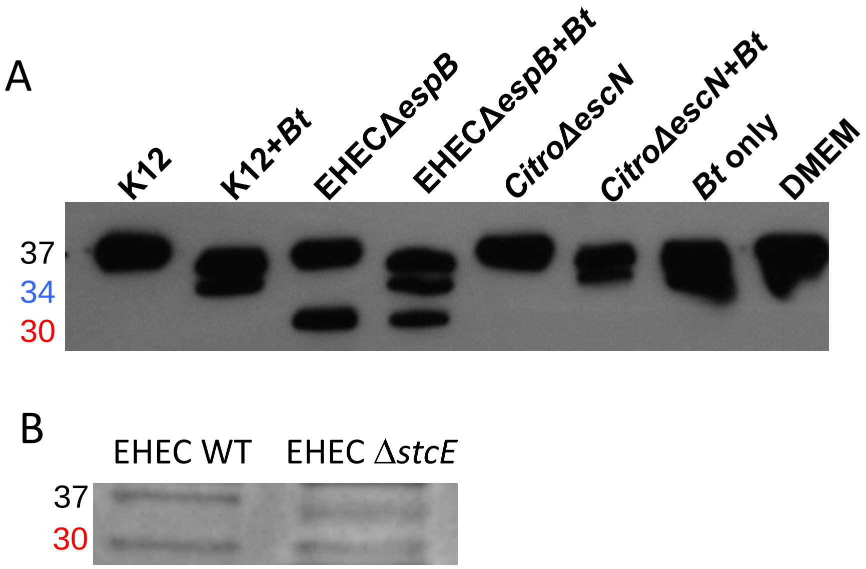

Fig.S2.EspP is the EHEC protease that cleaves EspB. (A) rEspB was incubated from supernatants from *E. coli* K12, *ΔespB* EHEC or a T3SS-deficient (*ΔescN) Citrobacter rodentium* strain +/- *B. theta.* (B) Western blot of endogenous EspB in WT and Δ*stcE* supernatants.
