# Supplementary material for: Microbiota and Pathogen Proteases Modulate Type III Secretion Activity in Enterohemorrhagic Escherichia coli
Source: mBio. 2018 Dec 4;9(6):e02204-18. doi: 10.1128/mBio.02204-18 (PMC6282197; doi:10.1128/mBio.02204-18)
Supplement: FIG S3 [file mbo006184200sf3.pdf]

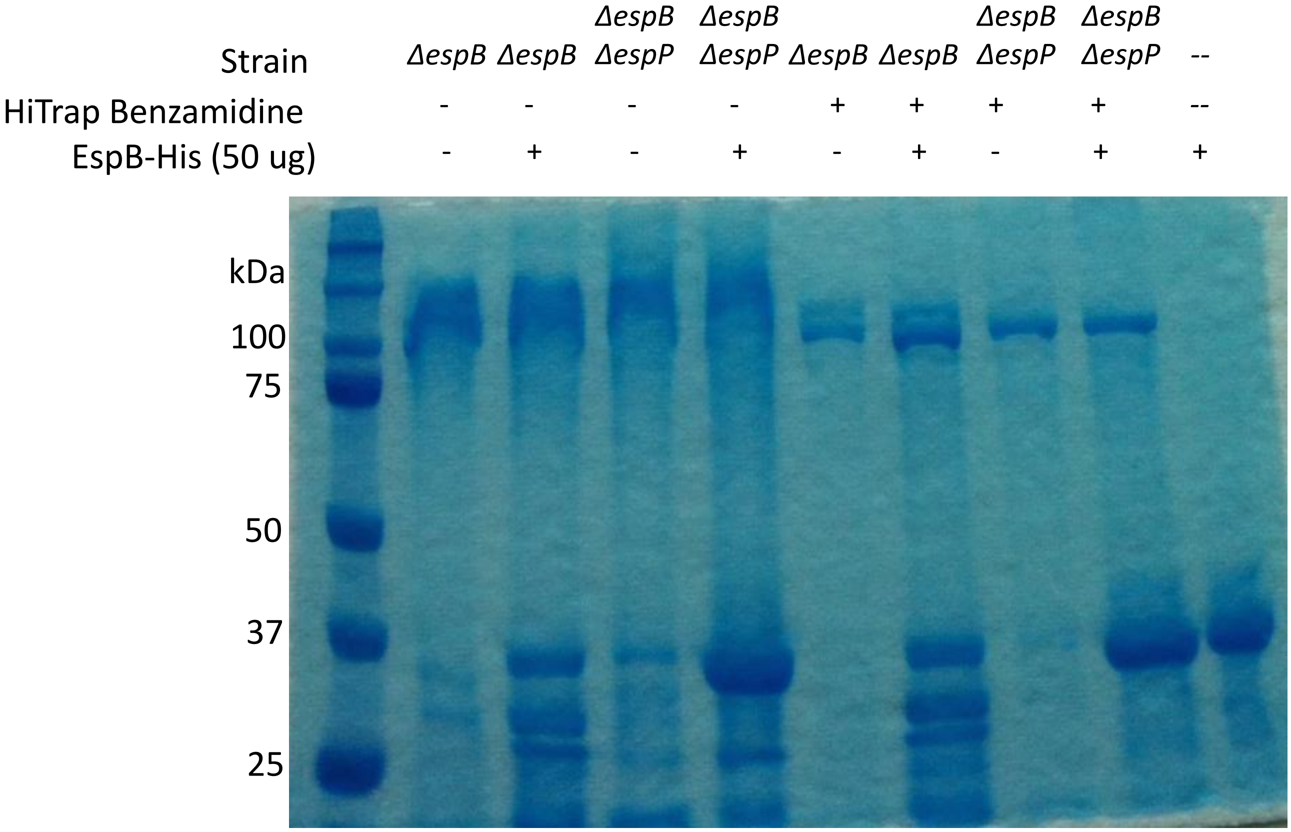

Fig. S3. Commassie stain of HiTrap Benzamidine trapping of EspP used for espB degradation.
Grown Δ*espB or* Δ*espB*Δ*espP* in 200 ml low-glucose DMEM, 37C, 5% CO_2 for 4.5 hrs; filtered and concentrated with 10 kDa cut-off to 1 ml; buffer-exchanged 500 ul with PBS; buffer-exchanged other 500 ul with Binding Buffer and ran over HiTrap Benzamidine column; eluted with 7.5 ml glycine buffer, pH 3.0 into 2 ml Tris buffer, pH 9.0 → buffer-exchanged with PBS to volume of 200 ul. Incubated samples +/- 50 ug of EspB overnight, 37°C; ran on 12% SDS-PAGE and Coomasie stained
