# Supplementary material for: Microbiota and Pathogen Proteases Modulate Type III Secretion Activity in Enterohemorrhagic Escherichia coli
Source: mBio. 2018 Dec 4;9(6):e02204-18. doi: 10.1128/mBio.02204-18 (PMC6282197; doi:10.1128/mBio.02204-18)
Supplement: FIG S5 [file mbo006184200sf5.pdf]

Bt proteins identified in Bt EHEC co-culture supernatant		
**Locus Tag**	**Description**	**Predicted Domains**
**BT_2479**	Iron-regulated protein A	Peptidase M75
**BT_2064**	Uncharacterized protein	None
**BT_0241**	Uncharacterized protein	None
**BT_4263**	GAPDH	
**BT_3742**	Uncharacterized protein	None
**BT_2753**	Putative transmembrane protein	OmpA-like β-barrel

Fig.S5. *Bt* proteins identified in *Bt* + EHEC co-culture by Mass spectrometry
