# Supplementary material for: Microbiota and Pathogen Proteases Modulate Type III Secretion Activity in Enterohemorrhagic Escherichia coli
Source: mBio. 2018 Dec 4;9(6):e02204-18. doi: 10.1128/mBio.02204-18 (PMC6282197; doi:10.1128/mBio.02204-18)
Supplement: FIG S6 [file mbo006184200sf6.pdf]

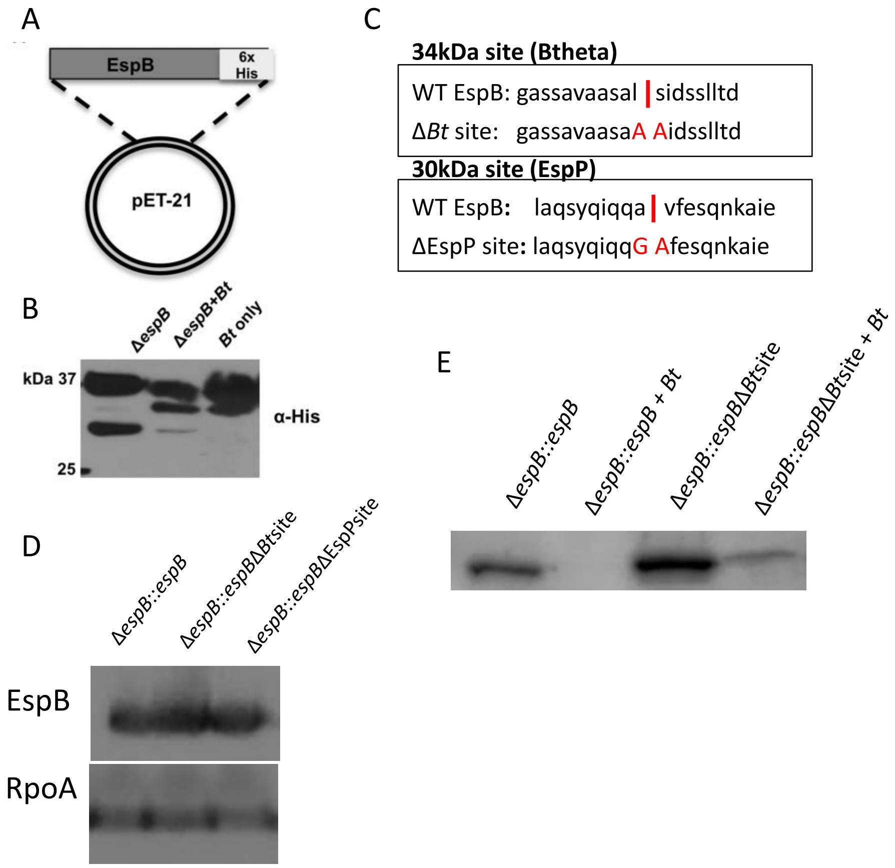

Fig. S6. Determination of EspB proteolysis sites.
(A) Schematic of pET21a-EspB-His. (B) Western blot analysis of exogenous rEspB-His using an anti-His antibody that will only detect cleaved EspB if the C-terminal His tag remains intact. (C) Schematic of site directed mutations made to the EspP and *Bt* protease cleavage sites. (D) Western blots of whole cell lysates collected from Δ*espB* EHEC complemented with either WT *espB, espB*Δ*Bt*site or *espB*ΔEspPsite in pACYC184. EspB appeared to be expressed to similar levels in all 3 strains, RpoA was used as a control for cell numbers. (E) Western blots of supernatants of EHEC Δ*espB* complemented with WT EspB or EspBΔBtsite cloned into vector pACYC184 in the absence and presence of *Bt* using anti-EspB antisera. There is a decrease in EspB cleavage in the EspBΔBtsite strain in the presence of Bt. However cleavage is not completely abrogated, this is probably due to the fact that the 36kDa *Bt* cleavage site is intact. We could only map the 34kDa site because we could not completely separate the 37 and 36kDa forms of EspB for cleavage analyses.
