# Supplementary material for: Microbiota and Pathogen Proteases Modulate Type III Secretion Activity in Enterohemorrhagic Escherichia coli
Source: mBio. 2018 Dec 4;9(6):e02204-18. doi: 10.1128/mBio.02204-18 (PMC6282197; doi:10.1128/mBio.02204-18)
Supplement: TABLE S2 [file mbo006184200st2.pdf]

Table S2. ***Strains and plasmids***

Strains		
Strain	*Genotype*	*Reference*
**EHEC**		
Wild-type (WT)	*E. coli* O157:H7 86-24 clinical isolate	(1)
Δ*espB* (MMC03)	isogenic *espB* deletion mutant	(2)
Δ*espA*	isogenic *espA* deletion mutant	This study
Δ*espD*	isogenic Δ*espD* deletion mutant	This study
Δ*espP*	isogenic Δ*espP* deletion mutant	This study
Δ*espB*Δ*espP*	isogenic Δ*espB*Δ*espP* deletion mutant	This study
Δ*espA*Δ*espP*	isogenic Δ*espA*Δ*espP* deletion mutant	This study
Δ*espD*Δ*espP*	isogenic Δ*espD*Δ*espP* deletion mutant	This study
WT p*bla*	WT 86-24 carrying *bla*:pBAD33	This study
WT *ptir-bla*	WT 86-24 carrying *tir-bla*:pBAD33	This study
Δ*espP* p*bla*	Δ*espP* 86-24 carrying *bla*:pBAD33	This study
Δ*espP ptir-bla*	Δ*espP* 86-24 carrying *tir-bla*:pBAD33	This study
Δ*espB*:: *espB*	Δ*espB* complemented with *espB*::pACYC184	This study
Δ*espB*:: *espB*Δ*Bt*site	Δ*espB* complemented with *espB*L31A S32A::pACYC184	This study
Δ*espB*:: *espB*ΔEspPsite	Δ*espB* complemented with *espB*A80G V81A::pACYC184	This study
Bacteroides thetaiotaomicron (Bt)		
VPI-5482	Wild-type, Gent^R	ATCC 29148
Citrobacter rodentium		
DBS770	Stx+ *C. rodentium*	*(3)*
MMC01	Δ*escN* isogenic deletion mutant of DBS770	*(4)*
E. coli		
MG1655	K12 *E. coli* strain	*E. coli* Genetic Stock Culture Collection; Yale University
Plasmids		
Plasmid	*Contents*	*Reference*
pKD3	λ red template plasmid	(5)
pKD46	λ red helper plasmid	(5)
pCP20	λ red helper plasmid	(5)
pDP151	Constitutive mCherry expression	
pACYC184	Cloning vector	New England Biolabs
pBAD33	Cloning vector with arabinose-inducible promoter	(6)
espB::pET21	*espB* from 86-24 in pET21a protein expression vector	This study
espA::pET28	*espA* from 86-24 in pET28a protein expression vector	This study
espD::pET21	*espD* from 86-24 in pET21a protein expression vector	This study
bla:pBAD33	*β-lactamse* under control of pBAD promoter in pBAD33	This study
tir-bla:pBAD33	*tir-β-lactamse* fusion under control of pBAD promoter in pBAD33	This study
espB::pACYC184	WT *espB* under the tet promoter in pACYC184	This study
*espB*Δ*Bt*site::pACYC184	*espB L31A S32A* under the tet promoter in pACYC184	This study
*espB*ΔEspPsite::pACYC184	*espB A80G V81A* under the tet promoter in pACYC184	This study

Supplement References

1. **Griffin PM, Ostroff SM, Tauxe RV, Greene KD, Wells JG, Lewis JH, Blake PA.** 1988. Illnesses associated with Escherichia coli O157:H7 infections. A broad clinical spectrum. Ann Intern Med **109:**705-712.
2. **Carlson-Banning KM, Sperandio V.** 2016. Catabolite and Oxygen Regulation of Enterohemorrhagic Escherichia coli Virulence. MBio **7**.
3. **Mallick EM, McBee ME, Vanguri VK, Melton-Celsa AR, Schlieper K, Karalius BJ, O'Brien AD, Butterton JR, Leong JM, Schauer DB.** 2012. A novel murine infection model for Shiga toxin-producing Escherichia coli. J Clin Invest **122:**4012-4024.
4. **Curtis MM, Hu Z, Klimko C, Narayanan S, Deberardinis R, Sperandio V.** 2014. The gut commensal Bacteroides thetaiotaomicron exacerbates enteric infection through modification of the metabolic landscape. Cell Host Microbe **16:**759-769.
5. **Datsenko KA, Wanner BL.** 2000. One-step inactivation of chromosomal genes in Escherichia coli K-12 using PCR products. Proc Natl Acad Sci U S A **97:**6640-6645.
6. **Guzman LM, Belin D, Carson MJ, Beckwith J.** 1995. Tight regulation, modulation, and high-level expression by vectors containing the arabinose PBAD promoter. J Bacteriol **177:**4121-4130.
