# Supplementary material for: Microbiota and Pathogen Proteases Modulate Type III Secretion Activity in Enterohemorrhagic Escherichia coli
Source: mBio. 2018 Dec 4;9(6):e02204-18. doi: 10.1128/mBio.02204-18 (PMC6282197; doi:10.1128/mBio.02204-18)
Supplement: TABLE S3 [file mbo006184200st3.pdf]

Table S3. ***primers***

**primer**	**Sequence 5'→3'**	**Description**
	qPCR	
rpoA F	GCGCTCATCTTCTTCCGAAT	housekeeping
rpoA R	CGCGGTCGTGGTTAGTG	housekeeping
espA F	TCAGAATCGCAGCCTGAAAA	
espA R	CGAAGGATGAGGTGGTTAAGCT	
espB F	TCAGATTCAGCAGGCCGTTT	
espB R	GTCACGGCAACAAATGCTGT	
espD F	TGTTGGCCAGGTCTTTGGTT	
espD R	TAACAGTGAAGACACCCCGC	
	Lambda Red mutagenesis	
espB lambda red F	AGCACGGGTAAATAGTCGTATTGTTAGTGGCCGAATTTAATTATTAAAGAGAATTTTATTGTGTAGGCTGGAGCTGCTTCG	EHEC Δ*espB* deletion
espB lambda red R	CCCTTTTTGTTCAAGATAGTAATTAAACACTTCATCATTAAACGTATCGACCATGATCAACATATGAATATCCTCCTTAG	EHEC Δ*espB* deletion
espD lambda red F	CAATCTCTACAATATAGAACTATTTCAGCAATATCCCTTGGTAAATAACCGGAGATAACTGTGTAGGCTGGAGCTGCTTCG	EHEC Δ*espD* deletion
espD lambda red R	AATTAACCATCGTTACTTGAGTATTATCAATAGTATTCATAATAAAATTCTCTTTAATAACATATGAATATCCTCCTTAG	EHEC Δ*espD* deletion
espP lambda red F	GTTTCTGAATTATCCGGCAGAGTATCATCAAGAGCAACTGGTAAGAAAAAACACAAACGGTGTAGGCTGGAGCTGCTTCG	EHEC Δ*espP* deletion
espP lambda red R	CAAAGGCGGATTTCTCAAACTCCAGTCCAAAGCGGACGTTATCCCTGATTTCTGCATTCCATATGAATATCCTCCTTAG	EHEC Δ*espP* deletion
	Plasmid construction	
espB::pET21 F	GGGGGGGGATCCATGAATACTATTGATAATACT	Recombinant EspB expression
espB:: pET21 R	CTCGAGCCCAGCTAAGCGAC	Recombinant EspB expression
espD::pET21 F	GGGGGGGGATCCATGCTTAACGTAAATAACGA	Recombinant EspD expression
espD::pET21 R	GCGGCCGCTAATTCGGCCACTAACAATA	Recombinant EspD expression
bla::pBAD gibson insert F	GGGCTAGGAAAAAGGAAGAGTATGAGTATTC	*bla*::pBAD33 construct for translocation assay
bla::pBAD gibson	CCAAGCTTGCTTACCAATGCTTAATCAGTGAGG	*bla*::pBAD33 construct

insert R		for translocation assay
bla::pBAD gibson vector F	GCATTGGTAAGCAAGCTTGGCTGTTTTGG	*bla*::pBAD 33 construct for translocation assay
bla::pBAD gibson vector R	CTCTTCCTTTTTCCTAGCCCAAAAAAACGGG	*bla*::pBAD 33 construct for translocation assay
tir-bla::pBAD gibson insert F	GGGCTAGAAAAGGAGATATTTATGCCTATTGGTAATCTTGGTC	*tir-bla*::pBAD 33 construct for translocation assay
tir-bla::pBAD gibson insert R	GCCAAGCTTGCTTACCAATGCTTAATCAGTG	*tir-bla*::pBAD 33 construct for translocation assay
tir-bla::pBAD gibson vector F	GCATTGGTAAGCAAGCTTGGCTGTTTTGG	*tir-bla*::pBAD 33 construct for translocation assay
tir-bla::pBAD gibson vector R	CAATAGGCATAAATATCTCCTTTTCTAGCCCAAAAAAACGGG	*tir-bla*::pBAD 33 construct for translocation assay
espB::pACYC184 gibson insert F	CCGTGTATGAATACTATTGATAATAC	*espB*::pACYC184 construct
espB::pACYC184 gibson insert R	GCTTCCATTTACCCAGCTAAGCG	*espB*::pACYC184 construct
espB::pACYC184 gibson vector F	GCTGGGTAAATGGAAGCCGG	*espB*::pACYC184 construct
espB::pACYC184 gibson vector R	CAATAGTATTCATACACGGTGCCTG	*espB*::pACYC184 construct

espB L31A S32A F	GCCGCATCTGCTGCAGCAATTGATTCATC	*espB* Δ*Bt* site mutagenesis
espB L31A S32A R	AACTGCACTGGAAGCGCCGG	*espB* Δ*Bt* site mutagenesis
espB A80G V81A F	CAGATTCAGCAGGGCGCTTTTGAGAGCCAG	*espB* ΔEspP site mutagenesis
espB A80G V81A F	ATAGCTTTGCGCCAATTGTTTTTG	*espB* ΔEspP site mutagenesis
